# The effect of pre-fracture proximal femur geometry on hip fracture type in elderly patients

**DOI:** 10.1097/MD.0000000000033622

**Published:** 2023-05-12

**Authors:** Mustafa Çukurlu, Bekir Karagoz, Ozan Keceli

**Affiliations:** a Adiyaman University Training and Research Hospital Department of Orthopaedics and Traumatology, Adiyaman, Turkey.

**Keywords:** fracture risk assessment, hip fracture, morphology, proksimal femur geometry

## Abstract

This study aimed to analyze the relationship between fracture type by determining data on the geometry of the proximal femur in the pre-fracture period in patients over 65 years of age who had hip fractures as a result of low-energy trauma. A total of 127 patients who were admitted to the hospital for reasons other than hip pathology within 1 year before the occurrence of hip fracture and who had an anterior-posterior pelvic X-ray were included in the study. Measurements were made to evaluate the proximal femur geometry, neck shaft angle, central edge angle, femoral head diameter, femoral neck diameter, femoral neck length, femoral offset length, femoral neck axial length, hip axis length, and femoral shaft diameter. As a result of these measurements, analyses were performed to determine the relationship between the control group and fracture types. The mean Neck shaft angle scores were significantly higher in both fracture types than in the control group (*P* = .034, *P* = .002). The mean Femoral offset length values of both fracture types were lower than those of the control group (*P* = .002, *P* = .011, respectively). Multiple logistic regression analysis revealed that the risk of collum femoris fracture increased as the Femoral head diameter value increased. (OD = 0.21, *P* = .002). The geometric parameters of the proximal femur play an important role in the formation of hip fracture types. Therefore, differences in proximal femur geometry in hip fracture types should be considered, and patient-focused choices should be made regarding the surgical procedures and implants to be used during fracture fixation.

## 1. Introduction

As the average age of the population has increased, hip fractures in the elderly have also become increasingly prevalent.^[1]^ Despite developments in orthopedic treatment techniques, it creates a serious burden on both society and the country’s economy, especially because it causes high mortality, limited mobility, and a decrease in quality of life.^[2,3]^ The types of hip fractures differ in terms of etiology, fall mechanism, bone mineral density, risk factors, patient characteristics, and morphological parameters of the proximal femur.^[4,5]^

The physical properties and geometric structures of objects are important factors that affect their strength.^[6]^ This was also the case for the proximal femur. Recent research has shown that the geometric structure of the bone is one of the key factors affecting the strength of the proximal femur.^[7,8]^ However, some studies have also shown that in patients with bilateral proximal femur fractures, the probability of fracture on both sides of the same anatomical location is 60% to 70%.^[9]^ Therefore, it is important to examine the geometric structures of the hip joint and proximal femur in patients with hip fractures.

The geometry of the proximal femur has been the subject of numerous studies in the literature.^[5,9,10]^ The characteristics of implants used in hip joint procedures and the effects of ethnic heterogeneity on proximal femoral geometry were the main topics of these studies. Additionally, studies examining the relationship between changes in proximal femur geometry and the type of fracture have been published.^[11,12]^ These studies compared data from the fractured side to the non-fracture side after including the non-fractured sides of patients with hip fractures. Considering the studies that found differences in the geometric structure of both hip joints of a healthy person, it is thought that comparing the non-fractured and fractured sides may affect the results.^[13]^ In our literature review, no study was found in which the effect of proximal femur geometry before fracture on the affected side on fracture types was examined. Our study emphasizes this deficiency and it is thought that the results obtained will contribute to the literature.

## 2. Material and methods

**Figure 1.**
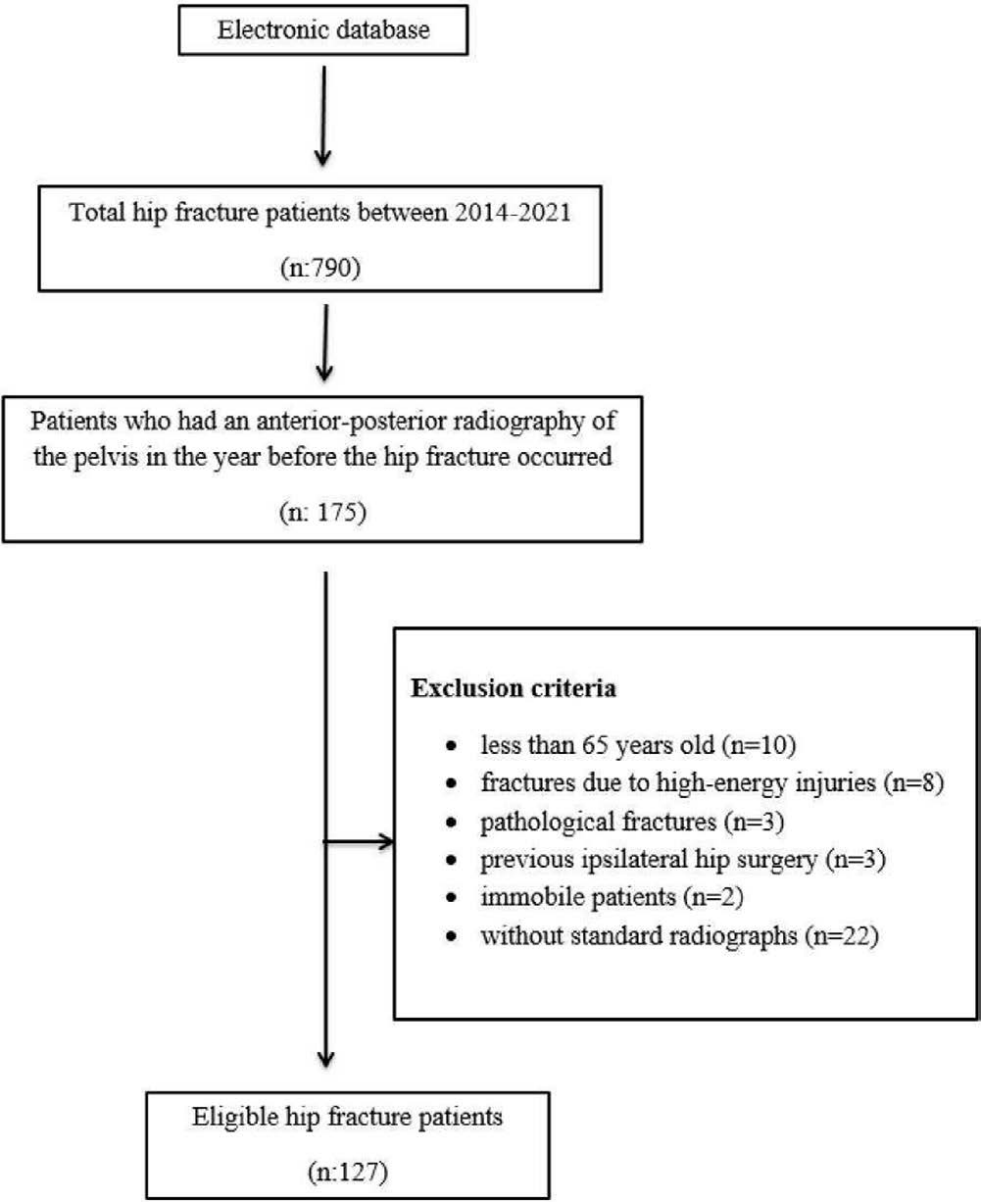
Flow chart of study design.

**Figure 2.**
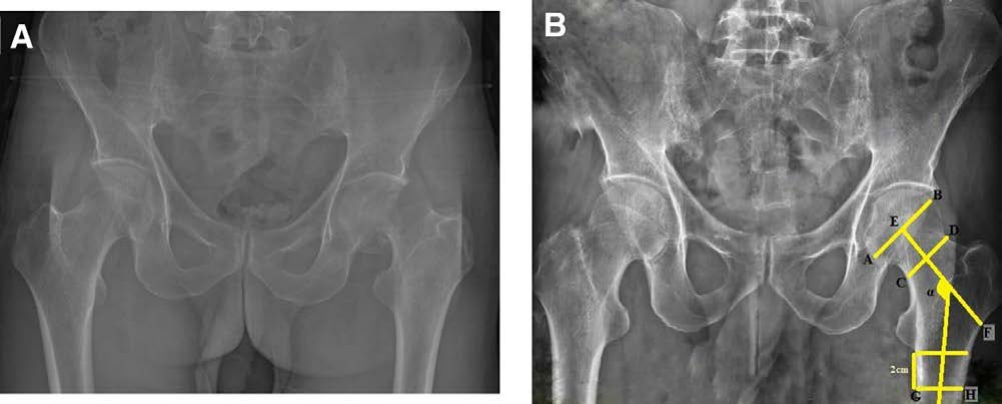
Anteroposterior radiograph of the pelvis shows the neck shaft angle (α), femoral head diameter (line AB), femoral neck diameter (line CD), femoral neck length (line EF), femoral shaft diameter (line GH) (B) and post-traumatic fracture radiograph of the same patient (A).

**Figure 3.**
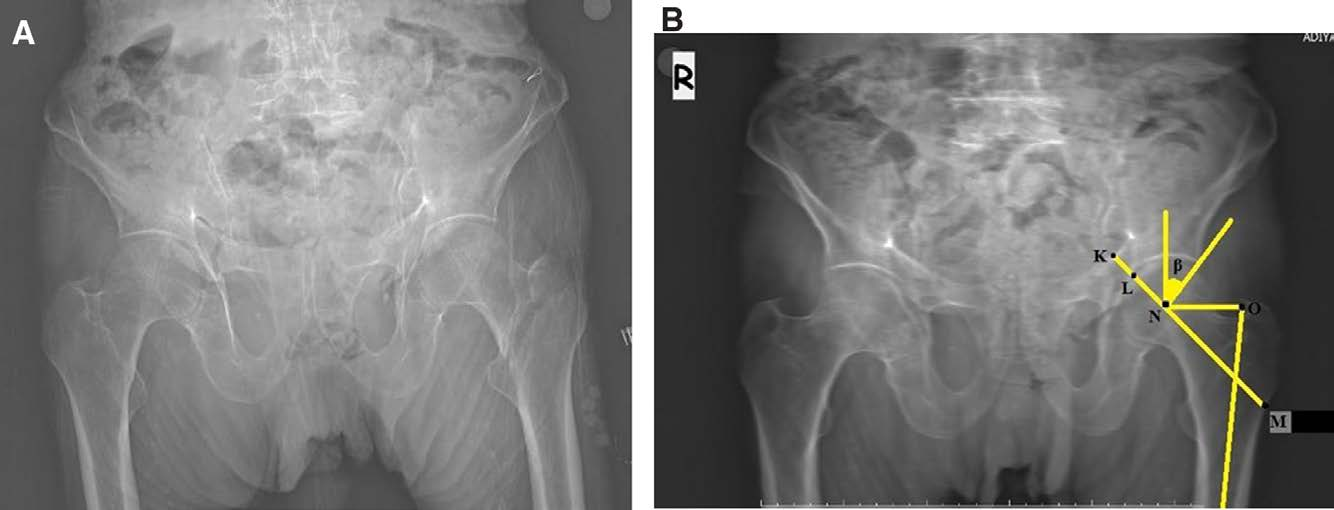
Anteroposterior radiograph of the pelvis shows the central edge angle (β), femoral offset length (line NO), femoral neck axial length (line LM), hip axis length (line KM) (B) and post-traumatic fracture radiograph of the same patient (A).

This retrospective study was approved by the medical ethics committee of Adiyaman University (2022/6–16). This study examined 790 consecutive patients admitted to our hospital with hip fractures between 2014 and 2021. It was determined that 175 of these patients were admitted to the hospital for reasons other than hip pathology within a year before the fracture occurred, and an anteroposterior (AP) radiograph of the pelvis was taken. Patients under the age of 65, those with fractures due to high-energy injuries, those with pathological fractures, those who had ipsilateral hip surgery, patients who were immobile, and those whose radiographs did not comply with the standards during the pre-fracture period were excluded from the study. The remaining 127 patients were enrolled in this study. The specific content is displayed in the flow chart Figure 1. Femoral neck fractures (Fig. 2A) were detected in 48 (37.8%) patients and intertrochanteric fractures (Fig. 3A) were found in 79 (62.2%) patients. The control group included in the study was randomly selected from patients over 65 years of age who had a pelvis AP radiograph taken in the hospital digital data system, no history of known hip pathology, and walked without support. A total of 100 patients (50 men, 50 women) who met these criteria were included in the control group.

Pelvic AP radiographs of the patients included in the study and the control group were obtained using the hospital’s digital data system, and a careful examination was performed. Pelvic AP radiographs were obtained in accordance with standards. Those with radiographs that did not comply with the standards were excluded from the study. Pelvic AP standard radiographs were obtained in the supine position, with the lower extremities parallel to each other, 15° to 20° internal rotation from the hip joint, and the knee in full extension, while the central radiographic ray was centered on the symphysis pubis.^[11]^ All pelvis AP radiographs were digitized using picture archiving communication system software. A digital goniometer with 1/1000 precision provided by the software and additional measuring instruments was used for all the required measurements. The following measurements were performed for the proximal femur geometric parameters:

The neck shaft angle (NSA) is the angle between the axis passing through the femoral shaft and the axis passing through the femoral neck (Fig. 2B).^[12]^

Central edge angle (CEA) is the angle between the vertical line passing through the center of hip rotation and the line drawn from the center of hip rotation to the outer edge of the acetabulum (Fig. 3B).^[14]^

The femoral head diameter (FHD) is the length corresponding to the diameter of the circle drawn on the femoral head on an AP radiograph of the pelvis (Fig. 2B).^[1]^

The femoral neck diameter (FND) is the shortest distance between the superior outer edge and the inferior inner edge of the femoral neck (Fig. 2B).^[1]^

Femoral neck length is the distance from the center of the femoral head to the axis of the femoral shaft, measured along the central axis of the femoral neck (Fig. 2B).^[11]^

Femoral offset length (FO) is the distance between the axis of the femoral shaft and the center of the femoral head (Fig. 3B).^[11]^

Femoral neck axial length is the distance along the central axis of the femoral neck between the apex of the femoral head and the lateral cortex of the femur (Fig. 3B).^[1]^

Hip axis length is the distance along the central axis of the femoral neck between the medial border of the pelvis and the lateral cortex of the femur (Fig. 3B).^[1]^

The femoral shaft diameter is the distance between the lateral and medial cortex of the femoral shaft perpendicular to the femoral shaft axis, 2 cm distal to the lower edge of the lesser trochanter (Fig. 2B).^[11]^

The relationships between the measurements, age, and fracture types were examined. analyses of fracture types were conducted in comparison with the control group. An experienced orthopedic and traumatology expert performed the measurements. The senior orthopedic and traumatology doctor in question repeated the measurements twice to evaluate intra-observer reliability. Three weeks after the first measurement, a second measurement was repeated. The intra-observer reliability of the measurements was determined at the 95% confidence interval by calculating the intra-class correlation coefficient, and was evaluated according to the İntra-class correlation coefficient interpretation of Landis and Koch (Table 1).^[15]^

**Table 1:** Intra-observer reliability analysis results.

Variables	ICC
NSA	0.975
CEA	0.963
FHD	0.995
FND	0.998
FNL	0.992
FO	0.879
FNAL	0.896
HAL	0.901
FSD	0.983

CEA = central edge angle, FHD = femoral head diameter, FNAL = femoral neck axial length, FND = femoral neck diameter, FNL = femoral neck length, FO = femoral offset length, FSD = femoral shaft diameter, HAL = hip axis length, ICC = Intra-class correlation coefficient, NSA = neck shaft angle.

### 2.1. Statistical analysis

The statistical package program Statistical Package for the Social Sciences (SPSS) 25.0 (IBM Corp, Armonk, NY), was used for statistical analysis. The Shapiro–Wilk test was used for normality tests of continuous variables. *T* tests were applied for the differences in normally distributed continuous variables in terms of categorical variable groups. The relationships between the classified variables forming the 2 × 2 crosstabs were investigated using the Fisher exact test. In addition, the multivariate logistic regression model was used to determine which measurement factors were risky based on the types of fractures that the patients had. In the statistical tests, the level of significance was set at *P* < .05.

## 3. Results

Table 2 displays the results obtained by comparing measurements of age, gender, and proximal femur geometry between both fracture types and the control group. Accordingly, no significant difference was found between the groups in terms of femoral neck length, femoral neck axial length, hip axis length, and femoral shaft diameter values. The NSA values were significantly higher in patients with femoral neck fractures and intertrochanteric fractures (*P* = .034, *P* = .002, respectively). While no difference was found between the CEA values of the control group and patients with intertrochanteric fractures, the mean CEA values of those with femoral neck fractures were statistically lower (*P* = .099 and *P* = .004, respectively). The FHD values in the control group were higher than the FHD values of patients with intertrochanteric fractures (*P* = .02). The study found no difference in the mean FHD values between those with femoral neck fractures and the control group.

**Table 2:** Comparison of age and proximal femoral parameters between groups.

Variables	Control group	Intertrochanteric fracture	*P* value *	Femoral neck fracture	*P* value †
Number of patients	100 (%44.1)	79 (%34.8)	-	48 (%21.1)	-
Gender (female/male)	50/50	54/25	-	28/20	-
Age (yr)	78.17 ± 6.81	79.67 ± 7.98	.176	77.42 ± 10.22	.597
NSA (degrees)	128.81 ± 4.24	130.73 ± 5.92	.034	131.47 ± 6.19	.002
CEA (degrees)	40.83 ± 4.80	39.56 ± 5.44	.099	37.54 ± 7.02	.004
FHD (cm)	5.25 ± 0.42	5.1 ± 0.43	.020	5.32 ± 0.55	.658
FND (cm)	3.59 ± 0.43	3.60 ± 0.43	.979	3.76 ± 0.53	.032
FNL (cm)	3.90 ± 0.53	3.83 ± 0.55	.387	3.93 ± 0.67	.967
FO (cm)	5.38 ± 0.59	5.11 ± 0.59	.002	5.09 ± 0.57	.011
FNAL (cm)	10.41 ± 0.84	10.26 ± 0.86	.219	10.45 ± 1.08	.826
HAL (cm)	12.15 ± 1.14	12.06 ± 0.96	.480	12.38 ± 1.20	.254
FSD (cm)	2.97 ± 0.34	3.04 ± 0.32	.450	3.12 ± 0.41	.098

Values are mean ± Standard Deviation.

CEA = central edge angle, FHD = femoral head diameter, FND = femoral neck diameter, FNL = femoral neck length, FO = femoral offset length, FNAL = femoral neck axial length, HAL = hip axis length, FSD = femoral shaft diameter, NSA = neck shaft angle.

* Patients with trochanteric fracture versus controls.

† Patients with femoral neck fracture versus controls.

While the FND averages in those with femoral neck fractures were significantly higher than those in the control group (*P* = .032), the study found no difference between those with intertrochanteric fractures and the control group. The mean FO values of the control group were higher than those of patients with both types of fractures (*P* = .002, *P* = .011, respectively).

Table 3 shows the results of the multiple logistic regression analysis performed on the geometric parameters of the proximal femur in patients with intertrochanteric and femoral neck fractures. The analyses revealed a statistically significant difference between FHD and fracture types. Accordingly, the higher the FHD value, the higher was the risk of femoral neck fracture (OD = 0.21, *P* = .002). The study observed that the parameters measured for other proximal femur geometries did not pose a significant risk in terms of fracture type.

**Table 3:** Risk factors of proximal femoral geometry parameters for femoral neck fractures and femoral intertrochanteric fractures by multiple logistic regression analysis.

Variables	OR	95% Cl	*P* values
NSA	1.07	0.97–1.17	.175
CEA	1.05	0.98–1.12	.205
FHD	0.21	0.08–0.56	.002
FND	0.99	0.24–4.09	.984
FNL	0.64	0.17–2.38	.509
FO	1.45	0.42–5.00	.555
FNAL	1.79	0.54–5.89	.341
HAL	0.47	0.16–1.36	.162
FSD	1.74	0.36–8.39	.488

CEA = central edge angle, CI = Confidence interval, FHD = femoral head diameter, FNAL = femoral neck axial length, FND = femoral neck diameter, FNL = femoral neck length, FO = femoral offset length, FSD = femoral shaft diameter, HAL = hip axis length, NSA = neck shaft angle, OR = Odds ratio.

## 4. Discussion

In this study, patients who had an AP radiograph of the pelvis within a year before the fracture occurred were identified and relevant analyses were conducted. This study examined the relationship between the types of hip fractures and geometric parameters of the proximal femur. This study identified 2 crucial findings. When the most important result was compared with the control group, we observed a statistically significant increase in NSA and shortening in FO in patients with hip fracture. Another important finding is that an increase in FHD is a risk factor for femoral neck fractures.

Factors affecting hip fracture type have long been a topic of interest in the scientific world. One of these elements is the geometrical characteristics of the proksimal femur. Numerous proximal femur geometry factors are associated with this problem.^[16,17]^ The NSA is one of the most studied geometric parameters of.^[5,11,14]^ Partanen et al^[18]^ showed that an increase in NSA is a risk factor for femoral neck fracture. In another important study, Rafferty et al^[19]^ found that the upper part of the femoral neck had a lower thickness than the lower part in patients with a high NSA. They claimed that this is a high risk situation for femoral neck fractures in individuals with increased NSA. In addition, many studies have found that increased NSA values pose a high risk of femoral neck fractures.^[5,14,20]^ However, it is possible to conduct studies with different results.^[11]^ In our study, NSA values were significantly higher in patients with hip fractures. However, no relationship was found between fracture type and NSA in the analysis of the fracture types. The increase in the NSA affects the femoral offset, and in this case, it can change the load arm and abductor moment arm acting on the pelvis. We hypothesized that this circumstance raises the risk of hip fracture in patients with a shift in load distribution.

CEA is used to assess hip joint stability. Literature data have shown that CEA is a risk factor for intertrochanteric fractures among hip fracture types.^[1,14]^ Yamauchi et al^[14]^ conducted an important study on risk factors. Accordingly, osteophytes resulting from hip osteoarthritis in elderly patients cause acetabular rim enlargement, which increases CEA levels. Owing to these osteophytes, the place of compression in the femoral neck shifts from proximal to distal at the time of fall. Thus, the momentary force of impact causes maximum weight on the more lateral bone area of the proximal femur, resulting in an intertrochanteric fracture. In this study, different results were obtained regarding the effect of CEA levels on fracture types. Compared with the control group, the CEA value was found to be significantly higher in femoral neck fractures, but no difference was found between intertrochanteric fractures. In the regression analysis, we found that CEA did not create any risk factors between the 2 fracture types. CEA can be affected by both the width of the acetabulum and number of age-related osteophytes. We believe that the severity of hip arthrosis in the patients included in this study is responsible for the emergence of this result.

The analysis of FND revealed one of the most significant findings of our investigation. Few studies have examined the connection between FND and fracture type, and none of them showed a meaningful relationship between the 2.^[1,5]^ As a result of the multiple logistic regression analysis performed in our study to assess the link between fracture type and FND, we established that an increase in FND is a risk factor for the occurrence of femoral neck fracture. As stated above, while there was no change in the thickness of the lower cortex in the femoral neck with age, there was a decrease in the thickness of the upper cortex. Therefore, increased FND may cause a fracture in the femoral neck region by causing more load on the neck during a fall.

When choosing the surgical procedure for proximal femoral fractures and the implant to be used, FND is a crucial geometric parameter. It has important clinical references for determining the number and type of screws to be used.^[1]^ Different results have been reported in studies comparing FND and fracture type.^[17,21]^ In a study conducted by Hu et al^[1]^, FND was found to be shorter in patients with intertrochanteric fractures than in the control group; in their multiple logistic regression analysis, they found that an increase in neck width was protective for intertrochanteric fractures. In our study, we found that the FND was significantly higher in patients with femoral neck fractures than in the control group. However, it has been observed that this is not a risk factor for femoral neck fractures.

Although the femoral offset is defined as the distance between the center of the femoral head and the anatomical axis of the femur, it is clinically important because it represents the biomechanical arm of the hip abductor muscles.^[22]^ It is especially used for the stability of hip joint arthroplasty and for determining leg length.^[23]^ As a result, research on femoral offset has typically concentrated on these issues. However, very few studies have examined the connection between the different types of fracture.^[11,24]^ In one of these studies, Yamauchi et al^[14]^ claimed that the length of the femoral offset was not associated with the fracture type. According to Im et al^[11]^, a short femoral offset is a risk factor for femoral neck fracture. In our study, we observed that the femoral offset was significantly lower in the patients with hip fractures than in the control group. We believe that the increase in NSA affects the femoral offset in patients with hip fracture, and thus, it is effective for hip fractures together with NSA.

Our study has some limitations. First, this was a retrospective study. This may have caused the risk of retrospective bias. Although high results were obtained in the intra-observer reliability analysis, another limitation was that the measurements were performed by a single person in the study. In our institution, a standard protocol is applied for AP radiography of the pelvis. However, we believe that this is also a limitation because small errors that can be made during imaging may affect the measurements. Future studies on this topic should analyze measures with high intra and inter-observer reliability coefficients and involve more subjects.

## 5. Conclusion

These results suggest that proximal femur geometric parameters may play an important role in hip fracture types. Therefore, differences in proximal femur geometry in hip fracture types should be considered, and patient-focused choices should be made regarding the surgical procedures and implants to be used during fracture fixation.

## Author contributions

**Conceptualization:** Mustafa Çukurlu .

**Data curation:** Mustafa Çukurlu, Bekir Karagoz.

**Formal analysis:** Bekir Karagoz.

**Methodology:** Bekir Karagoz.

**Resources:** Mustafa Çukurlu, Ozan Keceli.

**Software:** Ozan Keceli.

**Validation:** Ozan Keceli.

**Writing – original draft:** Mustafa Çukurlu.

**Writing – review & editing:** Mustafa Çukurlu, Bekir Karagoz.
